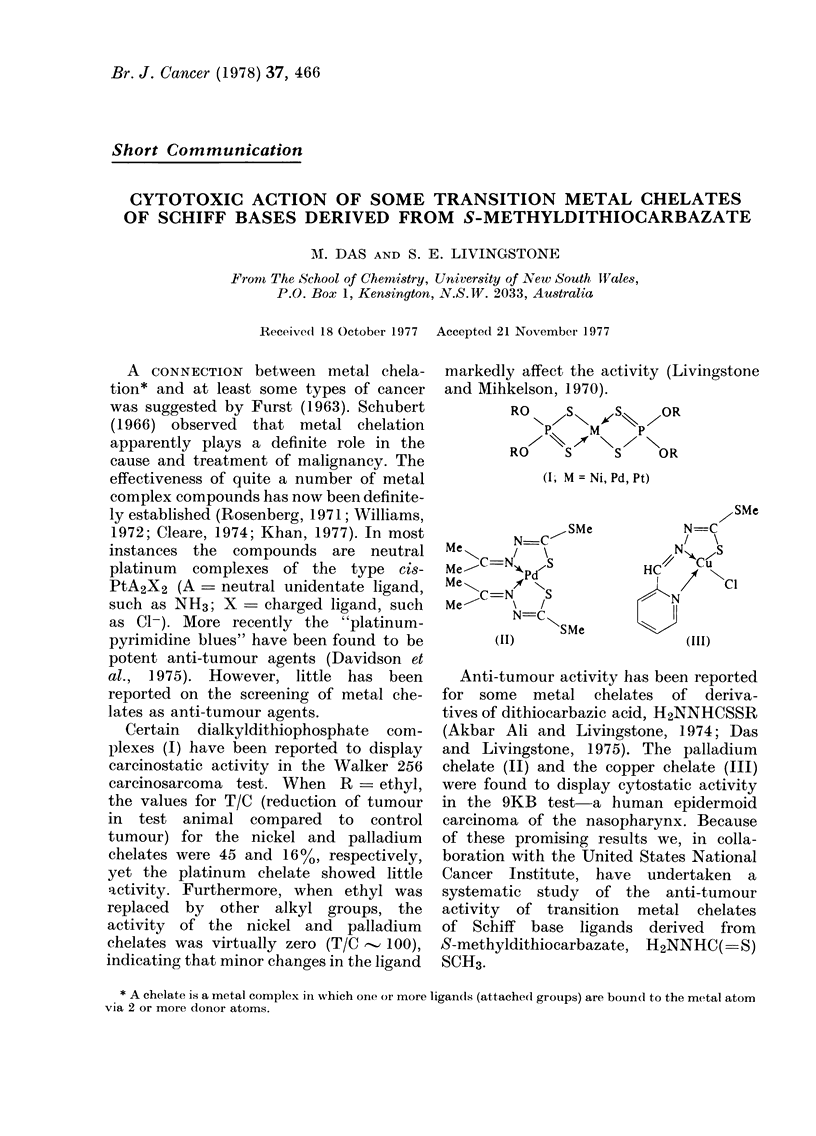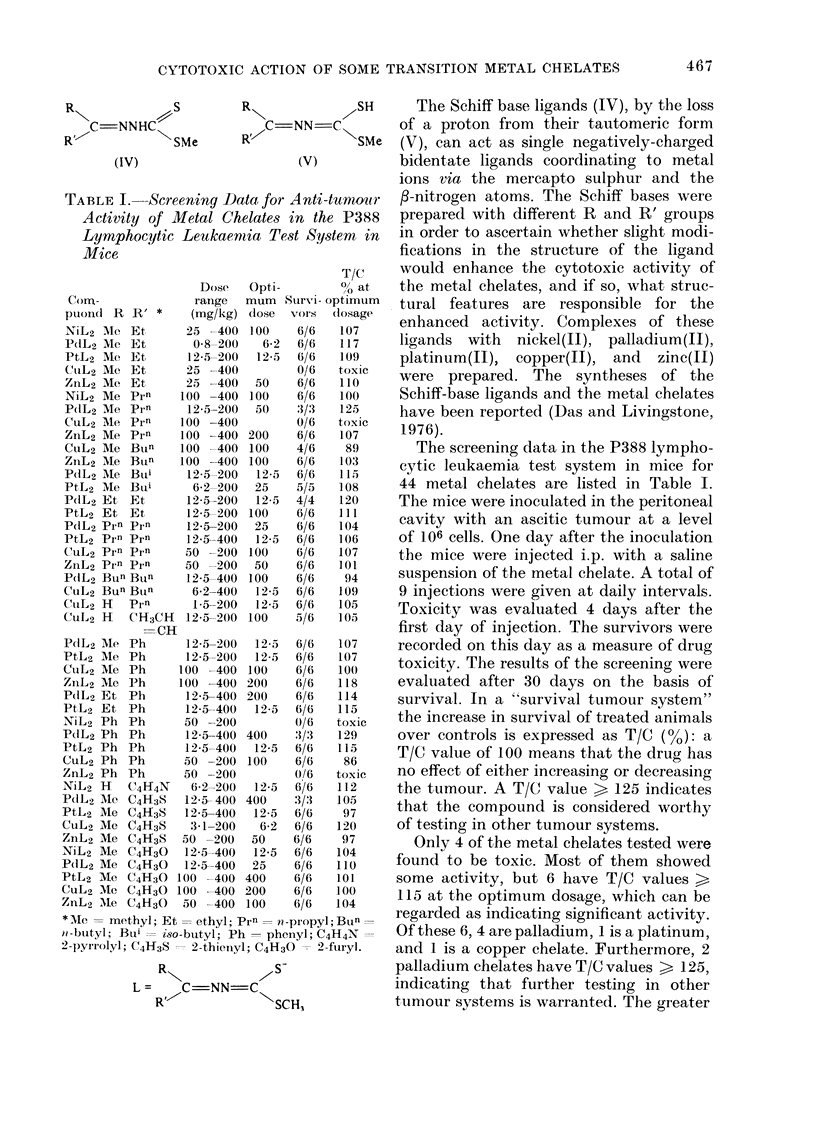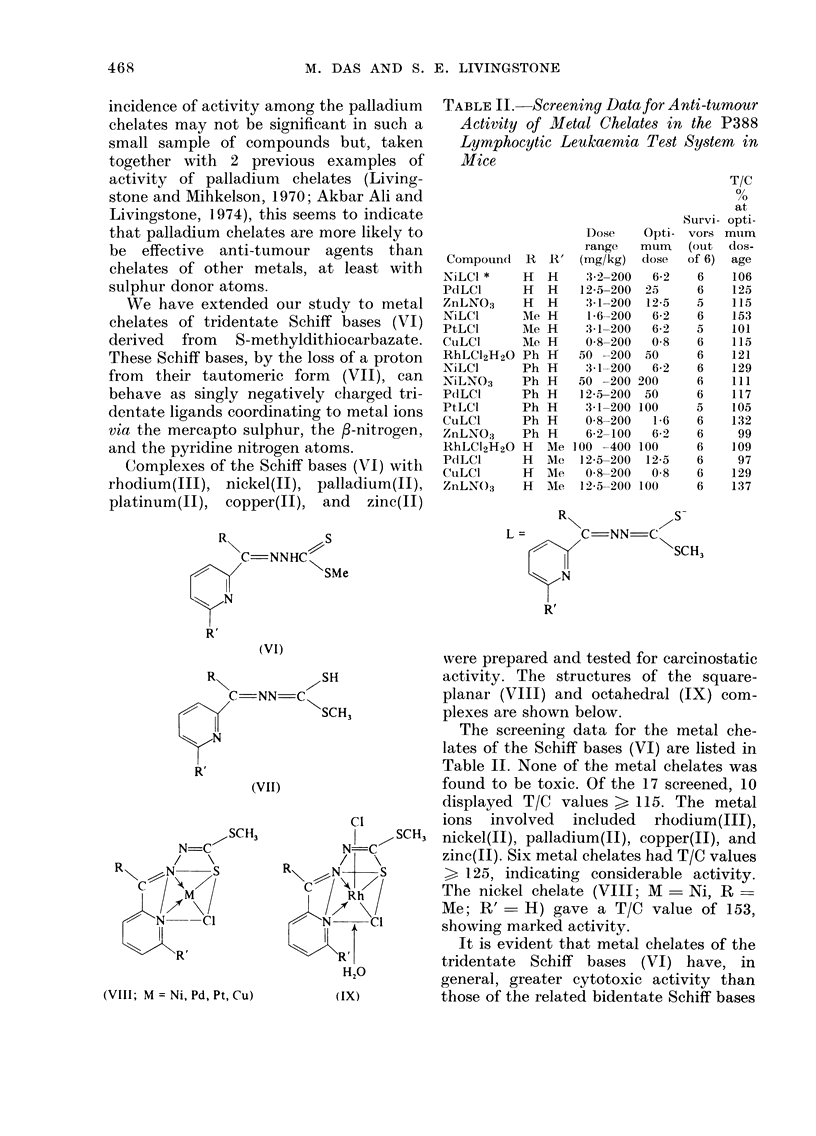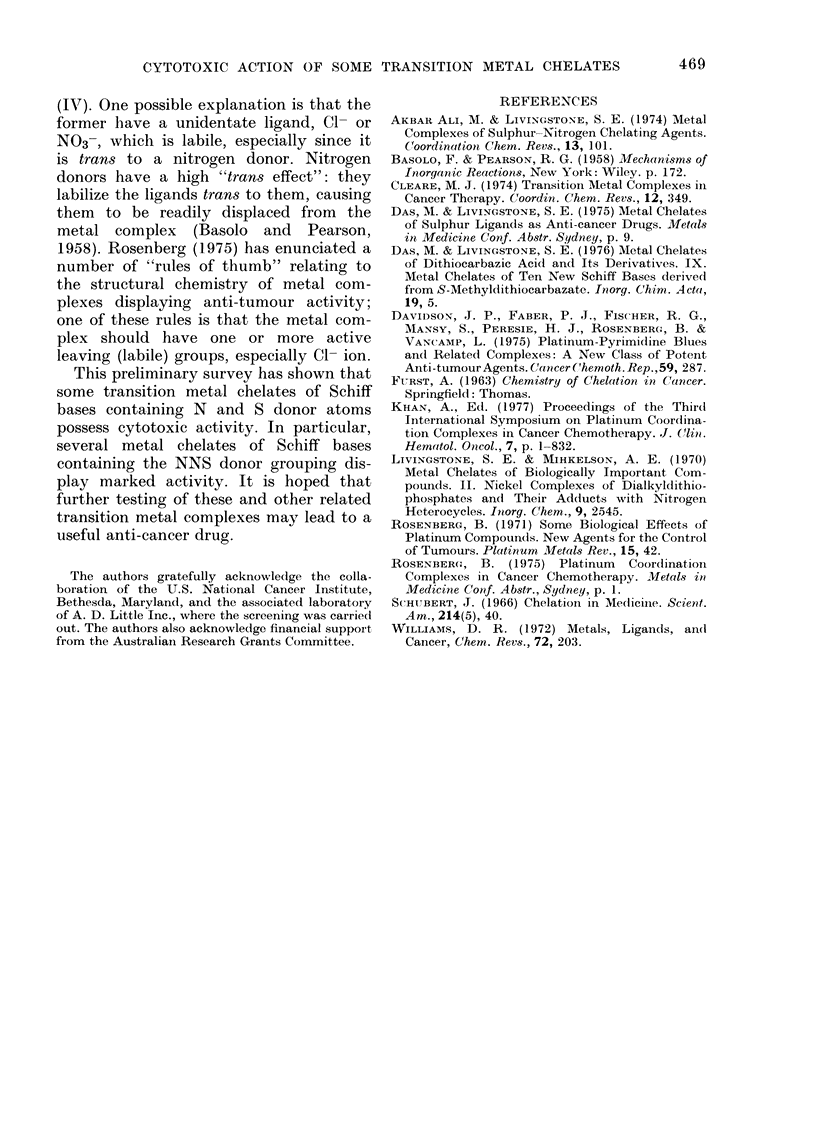# Cytotoxic action of some transition metal chelates of Schiff bases derived from S-methyldithiocarbazate.

**DOI:** 10.1038/bjc.1978.68

**Published:** 1978-03

**Authors:** M. Das, S. E. Livingstone


*Br. J. Cancer* (1978) **37**, 466

***Short Communication***

### CYTOTOXIC ACTION OF SOME TRANSITION METAL CHELATES OF SCHIFF BASES DERIVED FROM *S*-METHYLDITHIOCARBAZATE

M. DAS AND S. E. LIVINGSTONE

*From The School of Chemistry, University of New South Wales, P.O. Box 1, Kensington, N.S.W. 2033, Australia*

Received 18 October 1977 Accepted 21 November 1977

A CONNECTION between metal chelation* and at least some types of cancer was suggested by Furst (1963). Schubert (1966) observed that metal chelation apparently plays a definite role in the cause and treatment of malignancy. The effectiveness of quite a number of metal complex compounds has now been definitely established (Rosenberg, 1971; Williams, 1972; Cleare, 1974; Khan, 1977). In most instances the compounds are neutral platinum complexes of the type *cis*-$PtA_2X_2$ (A = neutral unidentate ligand, such as $NH_3$; X = charged ligand, such as $Cl^-$). More recently the "platinum-pyrimidine blues" have been found to be potent anti-tumour agents (Davidson *et al.*, 1975). However, little has been reported on the screening of metal chelates as anti-tumour agents.

Certain dialkyldithiophosphate complexes (I) have been reported to display carcinostatic activity in the Walker 256 carcinosarcoma test. When R = ethyl, the values for T/C (reduction of tumour in test animal compared to control tumour) for the nickel and palladium chelates were 45 and 16%, respectively, yet the platinum chelate showed little activity. Furthermore, when ethyl was replaced by other alkyl groups, the activity of the nickel and palladium chelates was virtually zero (T/C ~ 100), indicating that minor changes in the ligand markedly affect the activity (Livingstone and Mihkelson, 1970).

(I, M = Ni, Pd, Pt)

(II)

(III)

Anti-tumour activity has been reported for some metal chelates of derivatives of dithiocarbazic acid, $H_2NNHCSSR$ (Akbar Ali and Livingstone, 1974; Das and Livingstone, 1975). The palladium chelate (II) and the copper chelate (III) were found to display cytostatic activity in the 9KB test—a human epidermoid carcinoma of the nasopharynx. Because of these promising results we, in collaboration with the United States National Cancer Institute, have undertaken a systematic study of the anti-tumour activity of transition metal chelates of Schiff base ligands derived from *S*-methyldithiocarbazate, $H_2NNHC(=S)SCH_3$.

* A chelate is a metal complex in which one or more ligands (attached groups) are bound to the metal atom via 2 or more donor atoms.

(IV)

(V)

TABLE I.—*Screening Data for Anti-tumour Activity of Metal Chelates in the* P388 *Lymphocytic Leukaemia Test System in Mice*

| Com-puond | R | R′ * | Dose range (mg/kg) | Opti-mum dose | Survi-vors | T/C % at optimum dosage |
|---|---|---|---|---|---|---|
| $NiL_2$ | Me | Et | 25–400 | 100 | 6/6 | 107 |
| $PdL_2$ | Me | Et | 0·8–200 | 6·2 | 6/6 | 117 |
| $PtL_2$ | Me | Et | 12·5–200 | 12·5 | 6/6 | 109 |
| $CuL_2$ | Me | Et | 25–400 | | 0/6 | toxic |
| $ZnL_2$ | Me | Et | 25–400 | 50 | 6/6 | 110 |
| $NiL_2$ | Me | $Pr^n$ | 100–400 | 100 | 6/6 | 100 |
| $PdL_2$ | Me | $Pr^n$ | 12·5–200 | 50 | 3/3 | 125 |
| $CuL_2$ | Me | $Pr^n$ | 100–400 | | 0/6 | toxic |
| $ZnL_2$ | Me | $Pr^n$ | 100–400 | 200 | 6/6 | 107 |
| $CuL_2$ | Me | $Bu^n$ | 100–400 | 100 | 4/6 | 89 |
| $ZnL_2$ | Me | $Bu^n$ | 100–400 | 100 | 6/6 | 103 |
| $PdL_2$ | Me | $Bu^i$ | 12·5–200 | 12·5 | 6/6 | 115 |
| $PtL_2$ | Me | $Bu^i$ | 6·2–200 | 25 | 5/5 | 108 |
| $PdL_2$ | Et | Et | 12·5–200 | 12·5 | 4/4 | 120 |
| $PtL_2$ | Et | Et | 12·5–200 | 100 | 6/6 | 111 |
| $PdL_2$ | $Pr^n$ | $Pr^n$ | 12·5–200 | 25 | 6/6 | 104 |
| $PtL_2$ | $Pr^n$ | $Pr^n$ | 12·5–400 | 12·5 | 6/6 | 106 |
| $CuL_2$ | $Pr^n$ | $Pr^n$ | 50–200 | 100 | 6/6 | 107 |
| $ZnL_2$ | $Pr^n$ | $Pr^n$ | 50–200 | 50 | 6/6 | 101 |
| $PdL_2$ | $Bu^n$ | $Bu^n$ | 12·5–400 | 100 | 6/6 | 94 |
| $CuL_2$ | $Bu^n$ | $Bu^n$ | 6·2–400 | 12·5 | 6/6 | 109 |
| $CuL_2$ | H | $Pr^n$ | 1·5–200 | 12·5 | 6/6 | 105 |
| $CuL_2$ | H | $CH_3CH{=}CH$ | 12·5–200 | 100 | 5/6 | 105 |
| $PdL_2$ | Me | Ph | 12·5–200 | 12·5 | 6/6 | 107 |
| $PtL_2$ | Me | Ph | 12·5–200 | 12·5 | 6/6 | 107 |
| $CuL_2$ | Me | Ph | 100–400 | 100 | 6/6 | 100 |
| $ZnL_2$ | Me | Ph | 100–400 | 200 | 6/6 | 118 |
| $PdL_2$ | Et | Ph | 12·5–400 | 200 | 6/6 | 114 |
| $PtL_2$ | Et | Ph | 12·5–400 | 12·5 | 6/6 | 115 |
| $NiL_2$ | Ph | Ph | 50–200 | | 0/6 | toxic |
| $PdL_2$ | Ph | Ph | 12·5–400 | 400 | 3/3 | 129 |
| $PtL_2$ | Ph | Ph | 12·5–400 | 12·5 | 6/6 | 115 |
| $CuL_2$ | Ph | Ph | 50–200 | 100 | 6/6 | 86 |
| $ZnL_2$ | Ph | Ph | 50–200 | | 0/6 | toxic |
| $NiL_2$ | H | $C_4H_4N$ | 6·2–200 | 12·5 | 6/6 | 112 |
| $PdL_2$ | Me | $C_4H_3S$ | 12·5–400 | 400 | 3/3 | 105 |
| $PtL_2$ | Me | $C_4H_3S$ | 12·5–400 | 12·5 | 6/6 | 97 |
| $CuL_2$ | Me | $C_4H_3S$ | 3·1–200 | 6·2 | 6/6 | 120 |
| $ZnL_2$ | Me | $C_4H_3S$ | 50–200 | 50 | 6/6 | 97 |
| $NiL_2$ | Me | $C_4H_3O$ | 12·5–400 | 12·5 | 6/6 | 104 |
| $PdL_2$ | Me | $C_4H_3O$ | 12·5–400 | 25 | 6/6 | 110 |
| $PtL_2$ | Me | $C_4H_3O$ | 100–400 | 400 | 6/6 | 101 |
| $CuL_2$ | Me | $C_4H_3O$ | 100–400 | 200 | 6/6 | 100 |
| $ZnL_2$ | Me | $C_4H_3O$ | 50–400 | 100 | 6/6 | 104 |

*Me = methyl; Et = ethyl; $Pr^n$ = *n*-propyl; $Bu^n$ = *n*-butyl; $Bu^i$ = *iso*-butyl; Ph = phenyl; $C_4H_4N$ = 2-pyrrolyl; $C_4H_3S$ = 2-thienyl; $C_4H_3O$ = 2-furyl.

L = 

The Schiff base ligands (IV), by the loss of a proton from their tautomeric form (V), can act as single negatively-charged bidentate ligands coordinating to metal ions *via* the mercapto sulphur and the $\beta$-nitrogen atoms. The Schiff bases were prepared with different R and R′ groups in order to ascertain whether slight modifications in the structure of the ligand would enhance the cytotoxic activity of the metal chelates, and if so, what structural features are responsible for the enhanced activity. Complexes of these ligands with nickel(II), palladium(II), platinum(II), copper(II), and zinc(II) were prepared. The syntheses of the Schiff-base ligands and the metal chelates have been reported (Das and Livingstone, 1976).

The screening data in the P388 lymphocytic leukaemia test system in mice for 44 metal chelates are listed in Table I. The mice were inoculated in the peritoneal cavity with an ascitic tumour at a level of $10^6$ cells. One day after the inoculation the mice were injected i.p. with a saline suspension of the metal chelate. A total of 9 injections were given at daily intervals. Toxicity was evaluated 4 days after the first day of injection. The survivors were recorded on this day as a measure of drug toxicity. The results of the screening were evaluated after 30 days on the basis of survival. In a "survival tumour system" the increase in survival of treated animals over controls is expressed as T/C (%): a T/C value of 100 means that the drug has no effect of either increasing or decreasing the tumour. A T/C value $\geqslant$ 125 indicates that the compound is considered worthy of testing in other tumour systems.

Only 4 of the metal chelates tested were found to be toxic. Most of them showed some activity, but 6 have T/C values $\geqslant$ 115 at the optimum dosage, which can be regarded as indicating significant activity. Of these 6, 4 are palladium, 1 is a platinum, and 1 is a copper chelate. Furthermore, 2 palladium chelates have T/C values $\geqslant$ 125, indicating that further testing in other tumour systems is warranted. The greater incidence of activity among the palladium chelates may not be significant in such a small sample of compounds but, taken together with 2 previous examples of activity of palladium chelates (Livingstone and Mihkelson, 1970; Akbar Ali and Livingstone, 1974), this seems to indicate that palladium chelates are more likely to be effective anti-tumour agents than chelates of other metals, at least with sulphur donor atoms.

We have extended our study to metal chelates of tridentate Schiff bases (VI) derived from S-methyldithiocarbazate. These Schiff bases, by the loss of a proton from their tautomeric form (VII), can behave as singly negatively charged tridentate ligands coordinating to metal ions *via* the mercapto sulphur, the β-nitrogen, and the pyridine nitrogen atoms.

Complexes of the Schiff bases (VI) with rhodium(III), nickel(II), palladium(II), platinum(II), copper(II), and zinc(II)

(VI)

(VII)

(VIII; M = Ni, Pd, Pt, Cu)

(IX)

TABLE II.—*Screening Data for Anti-tumour Activity of Metal Chelates in the* P388 *Lymphocytic Leukaemia Test System in Mice*

| Compound | R | R′ | Dose range (mg/kg) | Optimum dose | Survivors (out of 6) | T/C % at optimum dosage |
|---|---|---|---|---|---|---|
| NiLCl * | H | H | 3·2–200 | 6·2 | 6 | 106 |
| PdLCl | H | H | 12·5–200 | 25 | 6 | 125 |
| $ZnLNO_3$ | H | H | 3·1–200 | 12·5 | 5 | 115 |
| NiLCl | Me | H | 1·6–200 | 6·2 | 6 | 153 |
| PtLCl | Me | H | 3·1–200 | 6·2 | 5 | 101 |
| CuLCl | Me | H | 0·8–200 | 0·8 | 6 | 115 |
| $RhLCl_2H_2O$ | Ph | H | 50 –200 | 50 | 6 | 121 |
| NiLCl | Ph | H | 3·1–200 | 6·2 | 6 | 129 |
| $NiLNO_3$ | Ph | H | 50 –200 | 200 | 6 | 111 |
| PdLCl | Ph | H | 12·5–200 | 50 | 6 | 117 |
| PtLCl | Ph | H | 3·1–200 | 100 | 5 | 105 |
| CuLCl | Ph | H | 0·8–200 | 1·6 | 6 | 132 |
| $ZnLNO_3$ | Ph | H | 6·2–100 | 6·2 | 6 | 99 |
| $RhLCl_2H_2O$ | H | Me | 100 –400 | 100 | 6 | 109 |
| PdLCl | H | Me | 12·5–200 | 12·5 | 6 | 97 |
| CuLCl | H | Me | 0·8–200 | 0·8 | 6 | 129 |
| $ZnLNO_3$ | H | Me | 12·5–200 | 100 | 6 | 137 |

L =

were prepared and tested for carcinostatic activity. The structures of the square-planar (VIII) and octahedral (IX) complexes are shown below.

The screening data for the metal chelates of the Schiff bases (VI) are listed in Table II. None of the metal chelates was found to be toxic. Of the 17 screened, 10 displayed T/C values $\geqslant$ 115. The metal ions involved included rhodium(III), nickel(II), palladium(II), copper(II), and zinc(II). Six metal chelates had T/C values $\geqslant$ 125, indicating considerable activity. The nickel chelate (VIII; M = Ni, R = Me; R′ = H) gave a T/C value of 153, showing marked activity.

It is evident that metal chelates of the tridentate Schiff bases (VI) have, in general, greater cytotoxic activity than those of the related bidentate Schiff bases

(IV). One possible explanation is that the former have a unidentate ligand, $Cl^-$ or $NO_3^-$, which is labile, especially since it is *trans* to a nitrogen donor. Nitrogen donors have a high "*trans* effect": they labilize the ligands *trans* to them, causing them to be readily displaced from the metal complex (Basolo and Pearson, 1958). Rosenberg (1975) has enunciated a number of "rules of thumb" relating to the structural chemistry of metal complexes displaying anti-tumour activity; one of these rules is that the metal complex should have one or more active leaving (labile) groups, especially $Cl^-$ ion.

This preliminary survey has shown that some transition metal chelates of Schiff bases containing N and S donor atoms possess cytotoxic activity. In particular, several metal chelates of Schiff bases containing the NNS donor grouping display marked activity. It is hoped that further testing of these and other related transition metal complexes may lead to a useful anti-cancer drug.

The authors gratefully acknowledge the collaboration of the U.S. National Cancer Institute, Bethesda, Maryland, and the associated laboratory of A. D. Little Inc., where the screening was carried out. The authors also acknowledge financial support from the Australian Research Grants Committee.

REFERENCES

AKBAR ALI, M. & LIVINGSTONE, S. E. (1974) Metal Complexes of Sulphur–Nitrogen Chelating Agents. *Coordination Chem. Revs.*, **13**, 101.

BASOLO, F. & PEARSON, R. G. (1958) *Mechanisms of Inorganic Reactions*, New York: Wiley. p. 172.

CLEARE, M. J. (1974) Transition Metal Complexes in Cancer Therapy. *Coordin. Chem. Revs.*, **12**, 349.

DAS, M. & LIVINGSTONE, S. E. (1975) Metal Chelates of Sulphur Ligands as Anti-cancer Drugs. *Metals in Medicine Conf. Abstr. Sydney*, p. 9.

DAS, M. & LIVINGSTONE, S. E. (1976) Metal Chelates of Dithiocarbazic Acid and Its Derivatives. IX. Metal Chelates of Ten New Schiff Bases derived from *S*-Methyldithiocarbazate. *Inorg. Chim. Acta*, **19**, 5.

DAVIDSON, J. P., FABER, P. J., FISCHER, R. G., MANSY, S., PERESIE, H. J., ROSENBERG, B. & VANCAMP, L. (1975) Platinum-Pyrimidine Blues and Related Complexes: A New Class of Potent Anti-tumour Agents. *Cancer Chemoth. Rep.*, **59**, 287.

FURST, A. (1963) *Chemistry of Chelation in Cancer.* Springfield: Thomas.

KHAN, A., Ed. (1977) Proceedings of the Third International Symposium on Platinum Coordination Complexes in Cancer Chemotherapy. *J. Clin. Hematol. Oncol.*, **7**, p. 1–832.

LIVINGSTONE, S. E. & MIHKELSON, A. E. (1970) Metal Chelates of Biologically Important Compounds. II. Nickel Complexes of Dialkyldithiophosphates and Their Adducts with Nitrogen Heterocycles. *Inorg. Chem.*, **9**, 2545.

ROSENBERG, B. (1971) Some Biological Effects of Platinum Compounds. New Agents for the Control of Tumours. *Platinum Metals Rev.*, **15**, 42.

ROSENBERG, B. (1975) Platinum Coordination Complexes in Cancer Chemotherapy. *Metals in Medicine Conf. Abstr., Sydney*, p. 1.

SCHUBERT, J. (1966) Chelation in Medicine. *Scient. Am.*, **214**(5), 40.

WILLIAMS, D. R. (1972) Metals, Ligands, and Cancer, *Chem. Revs.*, **72**, 203.